# Application of Intramedullary Calcar Support Plate and Lateral Locking Plate in Elderly Patients with Neer 3 and 4-Part Fractures of Proximal Humerus Through a Deltoid Splitting Approach

**DOI:** 10.1007/s43465-024-01098-3

**Published:** 2024-03-01

**Authors:** Yijun Wang, Liang Zhao, Can Liu, Kang Qin

**Affiliations:** Department of Shoulder and Elbow Surgery, Center for Orthopedic Surgery, The Third Affiliated Hospital of Southern Medical University, 183 Zhongshan Avenue West, Guangzhou, 510630 China

**Keywords:** Proximal humerus fracture, Intramedullary calcar support plate, Neer 3 and 4-part fracture, Deltoid splitting approach

## Abstract

**Background:**

The reduction and fixation of Medial humeral calcar is difficult in the treatment of elderly proximal humerus Neer 3 and 4-part fractures with a single lateral locking plate. Our study investigated the efficacy of an intramedullary calcar supporting plate combined with a lateral locking plate for the treatment of 3- and 4-part fractures of the proximal humerus in the elderly through a deltoid splitting approach.

**Methods:**

From June 2022 to December 2022, we treated six elderly patients with Neer 3 and 4-part fractures using proximal humeral intramedullary calcar support plate in combination with lateral locking plate through a deltoid splitting approach. Follow-up time was 6–12 months. Assessment indicators included fracture union, quality of reduction, and complication rate. The Constant-Murley score was used to record shoulder function at 6 months postoperatively.

**Results:**

All 6 patients showed fracture union and anatomic reduction. Constant-Murley score was 79.5 (70–90) at 6 months postoperatively. There was no incision non-healing, internal fixation failure, bone non-union or surgical site infection, secondary surgery, or death. Shoulder impingement occurred in 1 case.

**Conclusion:**

Proximal humeral intramedullary calcar support plate combined with lateral locking plate fixation through a deltoid splitting approach can effectively maintain fracture reduction, prevent inversion collapse of humeral head and internal fixation failure, and provide satisfactory clinical results at an early stage.

**Supplementary Information:**

The online version contains supplementary material available at 10.1007/s43465-024-01098-3.

## Introduction

Proximal humeral fractures (PHFs) are adults’ seventh most frequent fractures [1]. Several studies have shown that prevalence varies from 4 to 10% of all fractures [2]. A linear increase in the incidence is present in the elderly population (> 65 years) [3–5]. According to Neer classification, most nondisplaced proximal humerus fractures can be treated nonoperatively. However, displaced fractures require surgery. However, surgery is recommended for displaced and 3 or 4-part fractures. Commonly used surgical methods are closed reduction and intramedullary nailing (CRIF), open reduction and internal fixation (ORIF), hemiarthroplasty (HA) and reverse shoulder arthroplasty (RSA) [6]. Among them, the proximal humeral locking plate is widely used in the treatment of proximal humeral fractures. However, for unstable, varus-displaced complex fractures of the proximal humerus, especially for elderly Neer 3 and 4-part fractures, surgical fixation is still challenging, and there is still a high incidence of postoperative complications [7–10]. Several studies have reported that lack of medial support is a predictor of failure after surgical fixation of proximal humeral fractures [11–13]. The double-plate technique has been used to improve the quality and stability of the reduction of the medial humeral calcar [14–16]. But this method requires stripping of more soft tissues and the blood supply around the fracture is difficult to preserve. Wang et al*.* [17] used a combined axillary approach and an anterolateral approach for direct reduction and fixation of the medial and lateral humeral columns.

Treatment of proximal humerus fractures via the deltoid approach has the advantages of easy visualization of the greater tuberosity and preservation of the branches of the anterior circumflex humeral artery. Intramedullary plates are used clinically for some special types of fractures to better protect the surrounding blood supply without an additional incision [18–21]. In this study, we used intramedullary calcar support plates combined with lateral locking plates via the deltoid approach to treat six cases of elderly Neer 3 and 4-part fractures with a satisfactory short-term clinical outcome at a follow-up of 6–12 months.

## Materials and Methods

All experimental procedures were approved by the Ethics Committee of Guangdong Orthopedic Hospital. All aspects of this cohort of cases were conducted in accordance with the current version of the Declaration of Helsinki, the guidelines established by the International Conference on the Harmonization of Standardized Clinical Practice, and Chinese laws. All participants signed an informed consent form before registration.

Inclusion criteria: 1. Age ≥ 60; 2. Neer 3 and 4-part closed fractures.

Exclusion criteria: 1. Neer 1 and 2-part closed fracture; 2. Open fractures; 3. Old fractures (≥ 3 weeks); 4. Pathologic fractures; 5. Polytrauma; 6. Fractures with blood vessels or nerves requiring repair; 7. With severe shoulder osteoarthritis; 8. With Large and massive rotator cuff tears.

Data of patients with Neer 3 and 4-part fractures using proximal humeral intramedullary calcar support plate in combination with lateral locking plate through a deltoid splitting approach were analyzed retrospectively. All patients underwent a clinical examination to document their soft tissue condition and neurovascular status, particularly the axillary nerve. Radiologic evaluation included anteroposterior and lateral scapular X-rays of the shoulder, and fractures were classified according to the Neer classification. CT scans were performed on all patients for further evaluation of fractures. The basic data of these six cases are shown in Table 1. According to Neer classification, four cases were 3-part fractures, and two cases were 4-part fractures. All surgeries were performed by the Department of Shoulder and Elbow Surgery, The Third Affiliated Hospital of Southern Medical University.

**Table 1:** Demographic and clinical characteristics of the six patients

Cases	Gender	Age	BMI	Affected side	Mechanism of injury	Neer type	Basic illness	Intraoperative bleeding (ml)	Operation time (mins)	Postoperative hospitalization (days)
1	Female	73	28.3	Right	Fall	4	HT	350	190	1 2
2	Female	78	28.5	Left	Fall	3	DB, HT	200	160	9
3	Female	69	24.1	Right	Fall	4		250	148	6
4	Female	80	22.5	Left	fall	3	HT	200	130	6
5	Female	65	22.6	Right	Fall	3		150	140	7
6	Male	60	27.8	Right	Traffic accident	3	DB	200	142	5
Average	–	70.8	25.6	–	–	–	–	225.0	151.7	7.5

*DB* Diabetes, *HT* Hypertension

## Surgical Technique

Surgical methods: General anesthesia, beach chair lying position.

An anterolateral approach through the deltoid muscle. The incision was about 10 cm long. The anterior and middle bundles of the deltoid muscle were bluntly separated to expose the tuberosity fractures, and the vascular and nerve bundles composed of the axillary nerve and the posterior humeral circumflex artery and vein were separated and protected. The large and small tuberosities and attached tendonous tissues were fixed with pre-set sutures, and the lesser and greater tuberosities were retracted forward and backward, respectively to expose the medullary cavity between the humeral head and the greater and lesser tuberosities. Remove the humeral head, tuberosity and blood clots at the distal end of the fracture and the soft tissue embedded in the medullary cavity, and clearly reveal the fracture line of the medial calcar. The shape of the fracture line on the medial calcar side was observed, and the humeral head, and humeral shaft were anatomically reduced. A pre-bent 3-hole “T”-shaped locking plate was applied intramedullary to the calcar to fix the fractured end of the medial calcar. The medial calcar was well reset under X-ray fluoroscopy, the allogeneic freeze-dried femoral head was taken, trimmed into a bone block similar to the bone defect in the medullary cavity, and implanted in the proximal bone defect, and the large and small nodules were pulled with suture rings to reset the large and small nodules, Insert the 3-hole proximal humeral locking plate for temporary fixation. After the fluoroscopy is satisfactory, screws are driven to firmly fix the fracture fragments, and the large and small tuberosity fragments are firmly sutured to repair the rotator cuff tissue. The incision was flushed, a drainage tube was placed, the incision was closed, and the shoulder rest was abducted and fixed after the operation (Supplementary Fig. 2).

### Postoperative Care and Follow-Ups

Postoperative X-ray examination of the shoulder. On the second day after the operation, the drainage tube was removed and the shoulder joint was passively moved with the assistance of the doctor. Isometric contraction exercises and elbow joint functional exercises were started within 1 week after the operation. Functional exercises were performed under the guidance of a rehabilitation specialist 2 weeks after the operation. Active activity training was started 1 month after the operation. After the postoperative review of the front and side of the shoulder, weight-bearing was allowed after the formation of the callus was observed.

We recorded the duration of surgery, the number of fluoroscopies, and blood loss. Patients were followed up every month postoperatively to record the complications (incisional complications, deep infection, screw penetration through the glenohumeral joint, shoulder impingement, internal fixation failure, avascular necrosis (AVN) of the humeral head, and reduction loss).

### Radiographic Evaluation

Neer classification was determined from pre-operative radiographs. A fracture part is considered displaced if angulation exceeds 45°, or if the fracture is displaced by more than 1 cm [22]. Patients were followed up with postoperative X-rays to document the quality of fracture reduction, fracture union, and complications. Postoperative imaging evaluations were performed on shoulder X-rays, including fracture displacement distance and humeral neck-shaft angle (HNSA). Measurements were graded according to anatomic reduction, acceptable reduction and poor reduction [23] (Table 2). Grade Criteria: Overall anatomic reduction of fracture (all indicators reach anatomic reduction), approximate anatomic reduction (more than 1 parameter is non-anatomic, but no poor displacement), and fracture poor displacement group (1 or more parameters of poor displacement) [24–26].

**Table 2:** Evaluation of imaging parameters for reduction

Parameter	Reduction quality		
	Anatomic reduction	Reduction acceptable	Poor reduction
Head stem displacement	Anatomy	≤ 5 mm	> 5 mm
Neck-shaft angle	Anatomy, 120°–150°	Slight varus, 110°–120°	Eversion, > 150° or severe varus, < 110°
Greater tubercle moves up	Anatomy	≤ 5 mm	> 5 mm

### Shoulder Function Score

The Constant-Murley Score (CMS) [27] is a multi-item functional scale assessing pain (15 scores), activities of daily living (ADL, 20 scores), range of motion (ROM, 40 scores) and strength of the affected shoulder (25 scores). Its score ranges from 0 to 100 points, representing the worst and best shoulder function, respectively.

## Results

Six cases (5 females, 1 male; average age 70.8 years (60–80 years); 4 cases of right shoulder and 2 cases of left shoulder) with medial cortical defect were treated using intramedullary calcar support plate fixation and minimally invasive lateral locking plate fixation through a deltoid splitting approach. According to Neer classification, there were 4 of 3-part fracture and 2 of 4-part fracture. Quality of reduction, fracture union, complications and functional scores were recorded. The average postoperative hospitalization was 7.5 days (5–12 days) and average operative time was 151.7 min (130–190 min) (Table 1).

Postoperative follow-up ranged from 6 to 12 months. Anatomic reduction was achieved in all six patients. Shoulder impingement occurred in one patient who was performed subacromial injection therapy after fracture union. The average Constant-Murley score of at the final follow-up was 79.5 scores (70–90) (Table 3).

**Table 3:** Functional results of the last follow-up of six patients

cases	Humeral head reduction	Neck-shaft angle	Greater tuberosity reduction	Fracture union (weeks)	Forward flexion	Abduction	External rotation	Internal rotation	VAS pain score	CMS	complication
1	Anatomy	136	Anatomy	16	150°	110°	45°	L5	0	80	–
2	Anatomy	132°	Anatomy	16	100°	80°	20°	S5	2	70	–
3	Anatomy	135°	Anatomy	12	160°	120°	45°	L4	0	84	–
4	Anatomy	133°	Anatomy	14	110°	100°	30°	L5	2	73	–
5	Anatomy	130°	Anatomy	12	120°	100°	50°	L3	0	80	–
6	Anatomy	132°	Anatomy	12	170°	120°	60°	L3	0	90	–
Average	–	133°	–	13.7	135°	105°	41.7°	–	0.7	79.5	–

*CMS* Constant-Murley score

## Discussion

Various surgical methods are available for the treatment of proximal humeral fractures, including percutaneous pin fixation, plate fixation, intramedullary nail fixation, and joint replacement. Treating Neer 3 and 4-part fractures of the proximal humerus in the elderly is challenging [6]. Some studies have shown that non-anatomic reduction, lack of medial support in proximal humeral fractures treated with plates, and disruption of blood supply at the fracture site are predictive factors for fixation failure [28–30]. Satisfactory reduction and support of the medial aspect of the proximal humerus reduce the risk of postoperative varus displacement, humeral head subsidence, and loss of neck-shaft angle [31–35]. Therefore, achieving a satisfactory reduction and fixation of the medial calcar of the proximal humerus plays a crucial role in the surgical management of proximal humeral comminuted fractures.

We used the approach through the anterior and middle bundles of the split deltoid muscle to achieve reduction and internal fixation of the fracture. In a cadaveric study, Gardner et al*.* [36, 37] reported that the anterior circumflex vessel courses directly in line with the deltopectoral approach. They confirmed that the surgical approach through the anterior deltoid raphe preserves both the anterior and posterior vascular supply to the humeral head. Rouleau et al*.* [38] suggested that the deltoid splitting approach offers full exposure of the lateral proximal humerus and has less impact on the anterior humeral circumflex artery. Bhayana et al*.* [39] believed that the deltoid splitting approach has advantages in achieving reduction and protecting the blood supply at the fracture site in Neer 3 and 4-part fractures of the proximal humerus involving the greater tuberosity. In this research, the displaced greater tuberosity provided a natural corridor for exposing the medial proximal humerus. When the deltoid splitting approach was used, the fracture can be clearly visualized by retracting the greater and lesser tuberosity posteriorly and anteriorly respectively, after the blood clots were removed. The intramedullary calcar support plate could be easily fixed under direct vision. After that, the connection between the humeral stem and the humeral head has been substantially stabilized, and the reduction of the fracture with a bone graft and a greater tuberosity becomes much easier, thereby minimizing the separation of the surrounding soft tissues. In our study, anatomic reduction was achieved in all six cases. Schnetzke et al*.* [40] demonstrated that anatomic reduction with a locked plate significantly improved the clinical outcome of unstable and displaced proximal humeral fractures involving the anatomic neck (10 of 30 patients with anatomic reduction). Habib et al*.* [41] reported that an overall anatomic or near-anatomic fracture reduction rate is 79.4% (27/34). Based on our experience, after the medial calcar of proximal humerus is reducted and fixed under a straight view using the intramedullary calcar support plate, the reduction of the humeral head can be effectively maintained. Then, reduction and fixation of the greater and lesser tuberosities can be easily performed, which reduces the surgical difficulty and improves the quality of fracture reduction in Neer 3and 4-part fractures.

All six patients showed fracture union and satisfactory reduction during follow-up. To make the humeral head well-supported and to minimize its collapse, allografts were performed after the placement of intramedullary calcar support plates, which were freeze-dried humeral heads. From a biomechanical perspective, the intramedullary calcar support plate, allograft, and lateral locking plate were used together to stabilize and support the proximal humeral fractures effectively. Some studies have used large autogenous/allogeneic fibular grafts to treat complex proximal humeral fractures [32, 42, 43], reducing postoperative reduction loss and increasing the fracture union rate compared to the control group. Our results are consistent with these studies. The average Constant-Murley score at the final follow-up was 79.5 scores (range from 70 to 90).

**Fig. 1:**
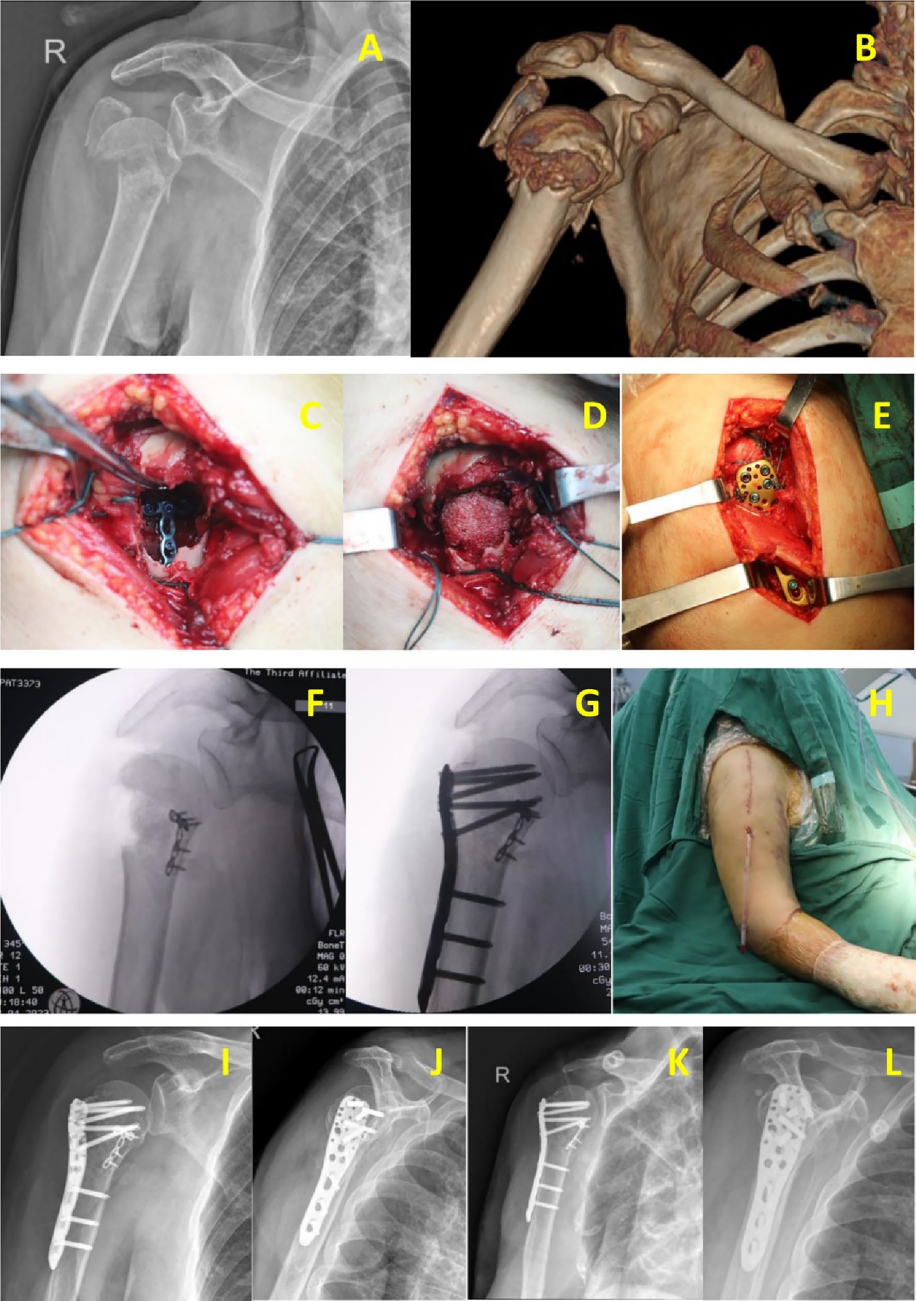
Typical case 1. A 73-year-old woman sustained an unstable proximal humerus fracture (Neer 3-part) from a fall. The pre-operative X-ray and CT scan showed severe displacement of proximal humerus (**A, B**); The medial supporting plate was used (**C**); An allogenic allograft was inserted in the bony defect (**D**); Protection of the axillary nerve (**E**); Intraoperative X-ray (**F, G**); Closure of the lateral approach (**H**). Radiographs 1 month after surgery (**I, J**); Radiographs 6 months after surgery (**K, L**)

**Fig. 2:**
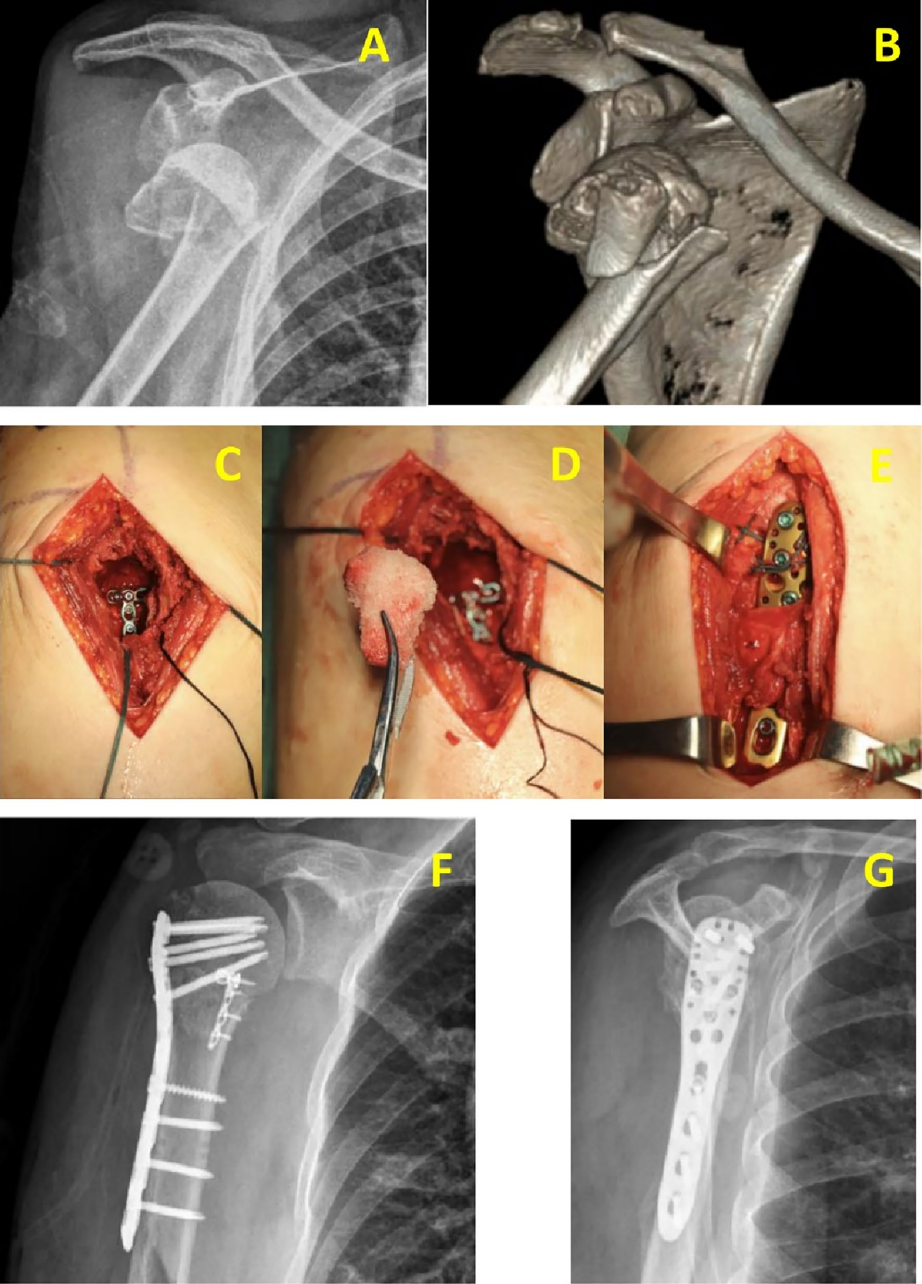
Typical case 2. An 80-year-old female patient suffered from right shoulder pain and limited mobility for 3 h due to a fall. The pre-operative X-ray and CT scan showed severe displacement of proximal humerus (**A, B**); The medial supporting plate was used (**C**); An allogenic allograft was inserted in the bony defect (**D**); Protection of the axillary nerve(**E**); Intraoperative X-ray (**F, G**)

Attempts have also been made to make fixation of proximal humerus fractures more stable and to allow patients early functional shoulder exercise. Shen et al*.* [33] used calcar screws to provide angular stability and maintain humeral head reduction, with an average Constant-Murley score of 80.25. Knierzinger et al*.* [44] have used angular stable plates and additional screw-tip cement augmentation to increase the stability of the internal fixation complex, with an average Constant-Murley score of 76 ± 15 at 1 year postoperatively, significantly reducing the internal fixation failure rate. Warnhoff et al*.* [15] used a double-plate technique, with a one-third tubular plate placed in front of the lesser tuberosity to maintain support of the medial humeral head, resulting in a Constant-Murley score of 77 ± 17. Wang et al*.* [16] added a support plate at the humeral tuberosity through the axillary approach and implanted an outer locking plate through the deltoid splitting approach, achieving a Constant-Murley score of 82.8. The scores in our study were similar to those studies above (Figs. 1, 2).

## Limitations

There are still several limitations in our study. First, this method is only applicable to Neer 3 and four-part fractures with clear displacement of the greater tuberosity, as it requires implantation of the intramedullary calcar support plate. Second, this is a retrospective study with a small number of cases and short follow-ups, which may not be sufficient to accurately assess the surgical long-term efficacy. Third, the biomechanical characteristics of the intramedullary calcar support plate have not been fully investigated. Therefore, we need to include more cases and perform further biomechanical studies, and design more scientific prospective controlled studies.

## Conclusion

Proximal humeral intramedullary calcar support plate combined with lateral locking plate fixation through a deltoid splitting approach can effectively maintain proximal humeral fracture reduction, and prevent inversion collapse and internal fixation failure, and the early clinical results are satisfactory. This may be a novel method for the elderly Neer 3 and 4-part fractures.

### Supplementary Information

Below is the link to the electronic supplementary material.

Supplementary file1 (DOCX 742 KB)

## Data Availability

Not applicable.
