# Supplementary material for: Application of Intramedullary Calcar Support Plate and Lateral Locking Plate in Elderly Patients with Neer 3 and 4-Part Fractures of Proximal Humerus Through a Deltoid Splitting Approach
Source: Indian J Orthop. 2024 Mar 1;58(4):362–70. doi: 10.1007/s43465-024-01098-3 (PMC10963710; doi:10.1007/s43465-024-01098-3)
Supplement: Supplementary file 1 — Supplementary file1 (DOCX 742 KB) [file 43465_2024_1098_MOESM1_ESM.docx]

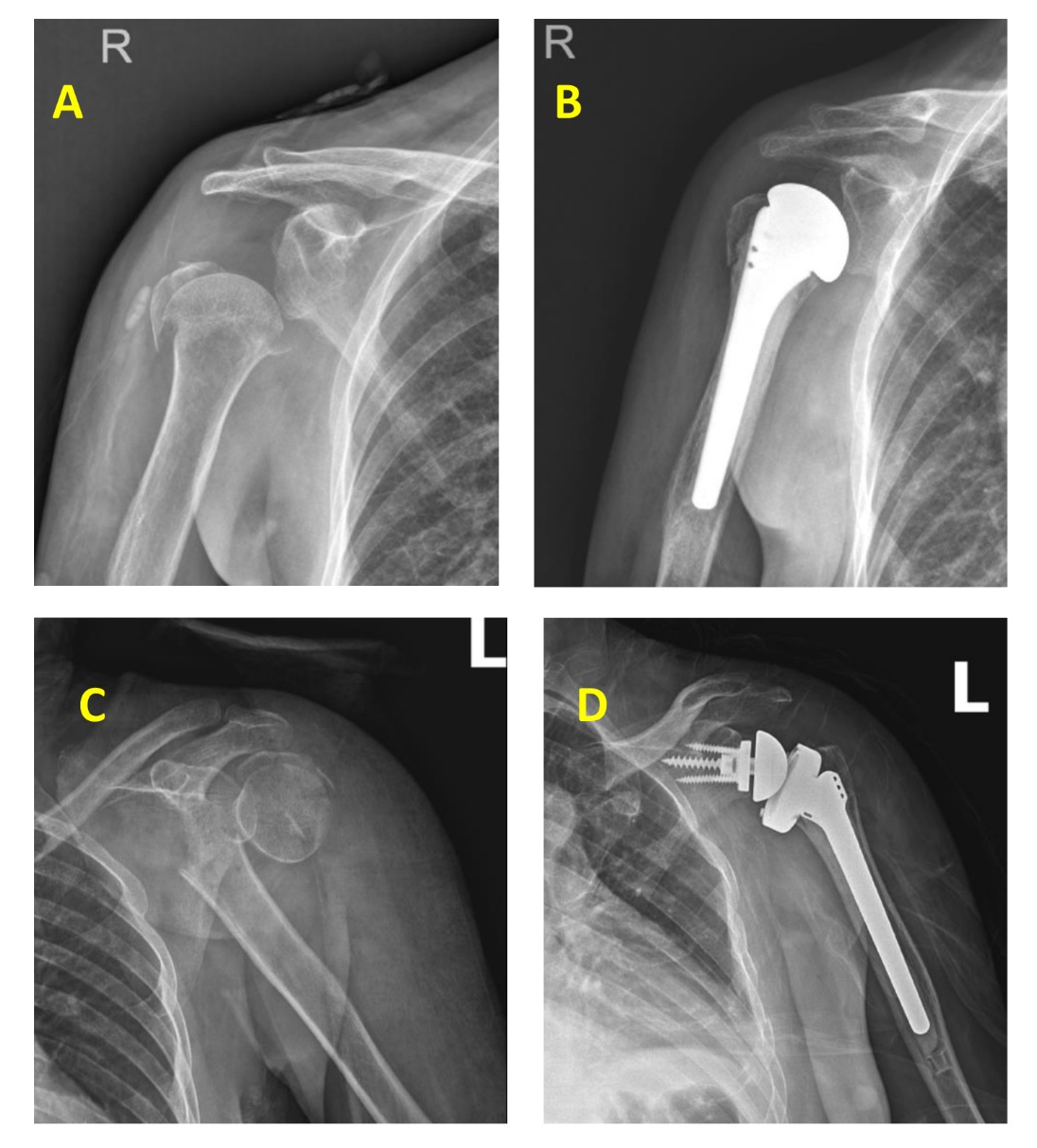


**Supplementary Figure 1.** (**A,B**) The pre- and post-operative X-rays of a 67-year-old female patient undergoing shoulder hemiarthroplasty. (**C,D**) The pre- and post-operative X-rays of a 70-year-old female patient undergoing reverse total shoulder replacement.


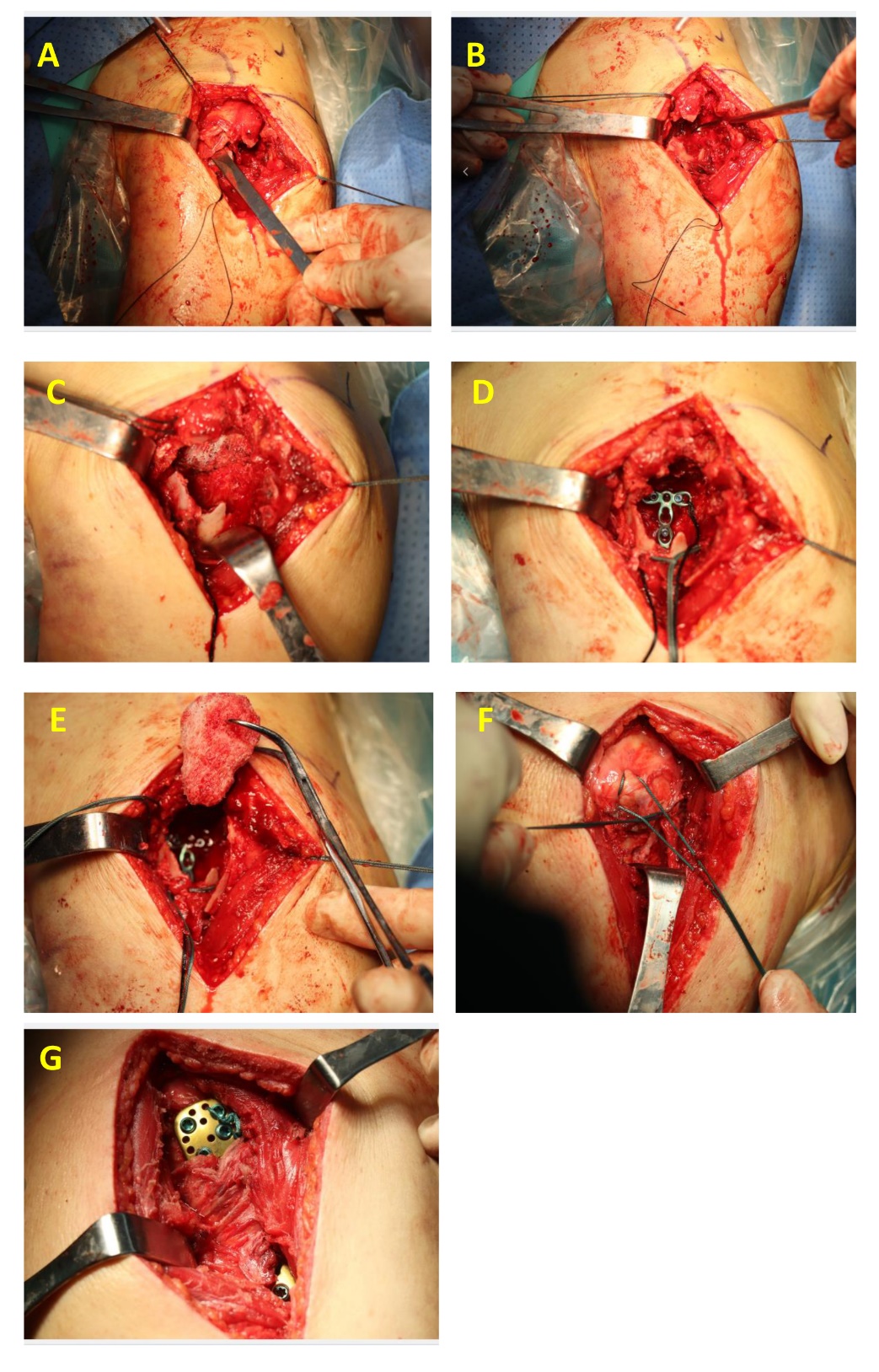


**Supplementary Figure 2. Surgical procedure (A**) Exposure of the greater tuberosity of the humerus; (**B**) Suture retraction of the greater/lesser tuberosity of the humerus; (**C**) Fracture line visible after removal of blood clots; (**D**) Placement of intramedullary calcar support plate; (**E**) Implantation of allograft bone into the bone marrow cavity of the proximal humerus; (**F**) Reduction of the greater/lesser tuberosity of the humerus; (**G**) Fixation of lateral locking plate.
